# Linkage of Australian national registry data using a statistical linkage key

**DOI:** 10.1186/s12911-021-01393-1

**Published:** 2021-02-02

**Authors:** Tim G. Coulson, Michael Bailey, Chris Reid, Gil Shardey, Jenni Williams-Spence, Sue Huckson, Shaila Chavan, David Pilcher

**Affiliations:** 1 Department of Epidemiology and Preventive Medicine, Monash University, Melbourne, Australia; 2 Centre for Integrated Critical Care, University of Melbourne, Melbourne, Australia; 3 Department of Anaesthesiology and Perioperative Medicine, Alfred Health, Melbourne, Australia; 4 Department of Medicine and Radiology, University of Melbourne, Melbourne, Australia; 5 The Australian and New Zealand Intensive Care Society (ANZICS) Centre for Outcome and Resource Evaluation (CORE), 277 Camberwell Road, Camberwell, VIC 3124 Australia; 6 The Department of Intensive Care, Alfred Health, Commercial Road, Prahran, VIC 3004 Australia; 7 School of Public Health, Curtin University, Perth, Australia

**Keywords:** Linkage, SLK-581, Registry

## Abstract

**Background:**

Data from clinical registries may be linked to gain additional insights into disease processes, risk factors and outcomes. Identifying information varies from full names, addresses and unique identification codes to statistical linkage keys to no direct identifying information at all. A number of databases in Australia contain the statistical linkage key 581 (SLK-581). Our aim was to investigate the ability to link data using SLK-581 between two national databases, and to compare this linkage to that achieved with direct identifiers or other non-identifying variables.

**Methods:**

The Australian and New Zealand Society of Cardiothoracic Surgeons database (ANZSCTS-CSD) contains fully identified data. The Australian and New Zealand Intensive Care Society database (ANZICS-APD) contains non-identified data together with SLK-581. Identifying data is removed at participating hospitals prior to central collation and storage. We used the local hospital ANZICS-APD data at a large single tertiary centre prior to deidentification and linked this to ANZSCTS-CSD data. We compared linkage using SLK-581 to linkage using non-identifying variables (dates of admission and discharge, age and sex) and linkage using a complete set of unique identifiers. We compared the rate of match, rate of mismatch and clinical characteristics between unmatched patients using the different methods.

**Results:**

There were 1283 patients eligible for matching in the ANZSCTS-CSD. 1242 were matched using unique identifiers. Using non-identifying variables 1151/1242 (92.6%) patients were matched. Using SLK-581, 1202/1242 (96.7%) patients were matched. The addition of non-identifying data to SLK-581 provided few additional patients (1211/1242, 97.5%). Patients who did not match were younger, had a higher mortality risk and more non-standard procedures vs matched patients. The differences between unmatched patients using different matching strategies were small.

**Conclusion:**

All strategies provided an acceptable linkage. SLK-581 improved the linkage compared to non-identifying variables, but was not as successful as direct identifiers. SLK-581 may be used to improve linkage between national registries where identifying information is not available or cannot be released.

## Background

There are an increasing number of clinical quality registries in Australia, with at least 40 currently in operation [1]. Some of these registries contain overlapping information, recording different sets of data for the same patients. Within these registries, some data will be duplicated, for example dates of admission, age, gender or treatment location. These duplicated data points may be leveraged to link between registries, enabling additional relevant information to be collected about patients and their outcomes and reducing costs associated with the data collection. By linking data between the databases we may gain insights into disease processes and risk factors that otherwise may not be considered, enabling the prediction of complications [2] or identification of new quality markers [3]**.**

Patient identifying data may vary according to the database or registry [4]. The gold standard for linkage would be linkage using name and address [5]. However, for historical and privacy reasons, some established registries do not have this information, or this data is held but privacy regulations restrict its release [6]. In some cases, data is collated with identifying information at the source hospitals, but identifiers are removed before transfer to the central registry. Data from these registries may be linked using other variables. These could include data such as dates of admission and discharge, age in years, sex or procedure dates. Errors may occur, primarily during data entry, often with single digit errors in the resulting database. This precludes matching accurately on all variables. Therefore, a hierarchy of matches using these variables, initially rigid, containing all variables and subsequently with reducing rigidity and variable number may be generated. As variable number reduces, the probability of achieving a match increases, although the probability of a mismatch also increases.

One way of handling privacy issues to improve linkage integrity without directly recording identifiable patient data is to introduce a statistical linkage key (SLK). This ‘key’ contains elements of unique patient identity without being directly linkable to the patient. One example, the SLK-581, was developed by the Australian Institute of Health and Welfare (AIHW). This is a 14-character code comprising the second, third and fifth characters of the family name, the second and third letters of the given name, the date of birth (DDMMYYYY) and sex [7]. This has shown to provide successful linkage in some datasets (for example a large residential aged care dataset), while its use has been less successful in others, particularly when a high rate of missing name data is present [8, 9]. SLK-581 has recently been added to the Australian and New Zealand Intensive Care Society Adult Patient Database (ANZICS-APD). It can also be added to other existing databases where identifying information is present. One such example is the Australian and New Zealand Society of Cardiothoracic Surgeons Cardiac Surgery Database (ANZSCTS-CSD). Given that almost all patients having cardiac surgery are admitted to intensive care, these two databases provide an opportunity to test SLK-581 linkage.

In this study our aim was to investigate the ability to link data using SLK-581 between the ANZICS-APD and ANZSCTS-CSD, and to compare this linkage to that achieved with direct identifiers (name and record number) or other non-identifying variables. Our hypothesis was that SLK-581 would improve linkage accuracy and reduce complexity.

## Methods

The ANZSCTS-CSD is a registry consisting of data related to all patients having cardiac surgery at participating centres in Australia and New Zealand. Patient identified data is submitted for the purpose of quality assurance and is stored securely. Only deidentified data is made accessible to third parties. The ANZICS-APD is a registry run by the ANZICS Centre for Outcome and Resource Evaluation. Deidentified data from adult ICUs throughout Australia and New Zealand are submitted for the purposes of benchmarking ICU performance and outcomes. Since Jan 2017 data submitted to ANZICS has contained the SLK-581. Both databases contain age, sex, dates of admission and discharge for hospital and ICU and unit identification (hospital).

The Alfred Hospital is a quaternary referral centre in Melbourne, which performs approximately 700 cardiothoracic operations per year. Data for submission to the ANZICS-APD is collected by dedicated trained data collectors. A deidentified dataset is then created at the site and submitted to ANZICS. In order to be able to use patient identifiers as a ‘gold standard’ of linkage we used data extracted from the ICU database of The Alfred Hospital for the purpose of submission to the ANZICS APD, but containing additional patient identifiers, prior to the data being deidentified and transferred to the central registry. Clinical data for submission to the ANZSCTS-CSD is collected primarily by medical staff from patient notes and then entered directly with patient identifiers (including name, medical record number, date of birth and address) onto a secure on-line data submission system by a data manager. SLK-581 was added to the ANZSCTS-CSD using the unique identifiers already present, and data was selected from the corresponding hospital (The Alfred Hospital) to match the ANZICS-APD data. Data was shared securely as clear text SLK-581 values. Ethical approval was granted by the Alfred Ethics Committee (approval number 86/19).

We analysed data from April 2017 to Dec 2018. We used the ANZSCTS-CSD as the primary data as it contained the patient admission episode related to the primary procedure and was less likely to contain duplicates, whereas patients could be admitted to ICU multiple times. Due to the fact that almost all cardiac surgical patients are admitted to ICU after their surgery, our aim was to match all cardiac surgical procedures (first admission only) to the subsequent ICU admission. Four strategies of matching were carried out.

### Strategy 1: using patient identifiers

To develop a ‘gold standard’ match we carried out probabilistic matching using surname, forename, medical record number (MRN) and SLK-581. To ensure admission episodes to ICU matched the surgical procedure an additional matching variable was added to the matching process using procedure date and intensive care admission date. These two dates had to match within 2 days. While the inclusion of ICU admission date/procedure date would likely reduce the number of matched patients, our aim was to match ICU admissions to their corresponding surgical procedure. A procedure date within 2 days of the ICU admission was deemed to be close enough to match the procedure with its corresponding ICU admission. Weights and cut-offs were assigned to variables and determined using multiple passes and clerical review to find the optimum weighting [5]. Clerical review examined all patients where exact matches in all fields were not generated and used other clinical data (for example, procedure type which is common to both datasets but not recorded in a standardised format) to check the accuracy of these matches.

### Matching weights and cut-offs were as follows

For the variables surname, forename and medical record number, positive weights were assigned of 7, 5 and 15, respectively. Negative weights were assigned of − 3, − 2 and − 5, respectively. A score for each match was determined based on these weights and a cutoff score of 1. For SLK-581 a positive weight of 10 and a negative weight of − 2 was assigned.

For procedure date/ICU admission date a positive weight of 2, a strongly negative weight of − 40 (such that non-matching procedure dates were rejected) and a caliper of 2 (to allow for admission within 2 days of the procedure) were assigned.

### Strategy 2: using deidentified variables only

To conduct matching without direct identifiers, we carried out a match using de-identified variables. This used 9 stages of matching and 6 variables (Table 1). Variables included in the match were age, sex, ICU admission/procedure date and discharge dates and hospital admission and discharge dates. Matching criteria for dates and ages were loosened to allow a discrepancy of ± 2 days (for dates) or years (for ages). If a pair of records were matched in stage 1 with tight criteria, they would also be matched when the criteria is loosened. Linking was sequential, so any patient that is matched in stage 1 was excluded from matching in stage 2 and so on. So, as the stages go up, the precision of the matching could drop slightly but so too does the number of possible matches. If enough deidentified variables are combined, the probability of having multiple patients match the criteria becomes very small.

**Table 1:** Matching stages using non identified data, with and without the SLK-581

Strategy 4: Matching stages using non-identifying variables and SLK-581	Strategy 2: Matching process using non-identifying variables without SLK-581
1. SLK-581 ICUAdmDt ICUDisDt HospAdmDt HospDisDt Age Sex	1. ICUAdmDt ICUDisDt HospAdmDt HospDisDt Age Sex
2. SLK-581 ICUAdmDt ICUDisDt HospAdmDt	2. ICUAdmDt ICUDisDt HospAdmDt HospDisDt Age
3. SLK-581 ICUAdmDt ICUDisDt	3. ICUAdmDt ICUDisDt HospAdmDt HospDisDt Sex
4. SLK-581 ICUAdmDt	4. ICUAdmDt ICUDisDt HospAdmDt Age Sex
5. ICUAdmDt ICUDisDt HospAdmDt HospDisDt Age Sex	5. ICUAdmDt ICUDisDt HospDisDt Age Sex
6. ICUAdmDt ICUDisDt HospAdmDt HospDisDt Age	6. ICUAdmDt HospAdmDt HospDisDt Age Sex
7. ICUAdmDt ICUDisDt HospAdmDt HospDisDt Sex	7. ICUDisDt HospAdmDt HospDisDt Age Sex
8. SLK-581 ICUAdmDt ICUDisDt HospAdmDt HospDisDt Sex	8. ICUAdmDt ICUDisDt HospAdmDt HospDisDt
9. SLK-581 ICUAdmDt ICUDisDt HospAdmDt Age Sex	9. ICUAdmDt ICUDisDt HospAdmDt
10. SLK-581 ICUAdmDt ICUDisDt HospDisDt Age Sex	
11. SLK-581 ICUAdmDt HospAdmDt HospDisDt Age Sex	
12. SLK-581 ICUDisDt HospAdmDt HospDisDt Age Sex	

*ICUAdmDt* ICU admission date/procedure date, *ICUDisDt* ICU discharge date, *HospAdmDt* Hospital admission date, *HospDisDt* Hospital discharge date

### Strategy 3: using SLK-581 and procedure date only

A match between the two databases was carried out using SLK-581 and the ICU admission/procedure date alone (allowing a discrepancy of 2 days).

### Strategy 4: using deidentified variables with SLK-581

We carried out a final match using the deidentified variables with SLK-581. This match used the same matching variables as strategy 2, with addition of SLK-581 (Table 1).

The rate of correct or incorrect matches was compared between the ‘gold standard’ using direct identifiers, and the other match strategies. We compared baseline characteristics (age, sex, procedure type, procedure urgency and risk of death within 30 days of cardiac surgery) between matched and unmatched patients for strategy 1. We then compared the same characteristics between unmatched patients in the 4 strategies.

Probabilistic matching was carried out using Stata Version 16 and the user written software ‘dtalink’ (strategy 1 and 3) [10]. SAS Version 9.4 (SAS Institute Inc., Cary, NC, USA) was used for deterministic matching (strategy 2 and 4) [11]. Chi square tests were used to compare match rates in different groups and for other categorical comparisons. The Student’s t-test was used to compare parametric data in two groups and ANOVA was used for multiple groups comparisons. Non-normally distributed data was compared using Wilcoxon Rank Sum (two groups) or Kruskal–Wallis tests (multiple groups).

## Results

Data from the hospital ICU data contained 5179 ICU admissions (for all medical and surgical admissions) from April 1st 2017 to December 31st 2018. There were a total of 1283 patient procedures contained within the corresponding ANZSCTS-CSD. 47/5179 patients in the ICU database had missing medical record number and names due to data error. Patients who had missing direct identifiers still had SLK-581 available. All information submitted to ANZSCTS-CSD had identifying information available.

### Strategy 1

**Fig. 1:**
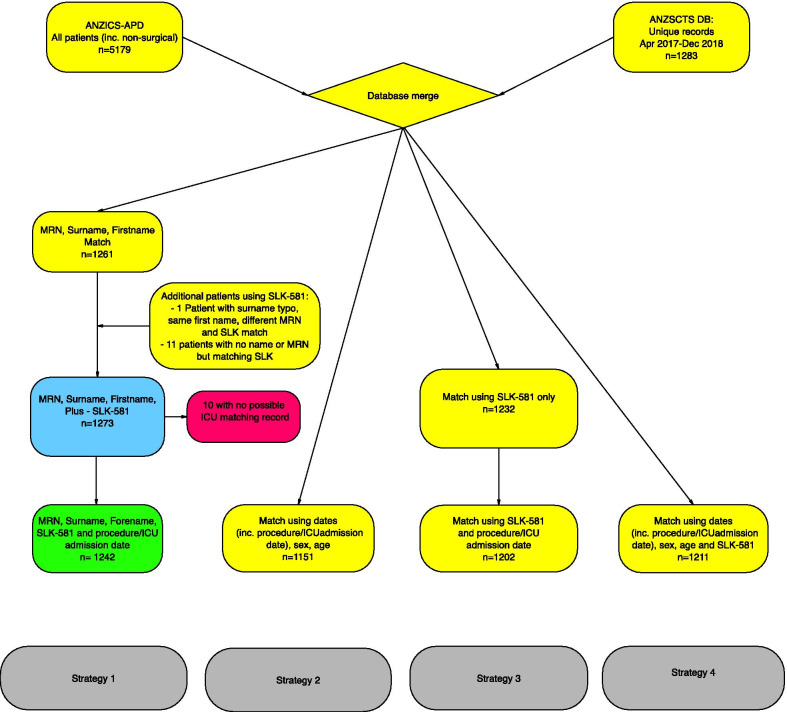
Results of matching using 4 different methods. The green box represents the ‘gold standard’ match, where procedure dates were matched to admission dates. The bottom row shows matches between both patient details and their ICU admission immediately subsequent to surgical procedure (and therefore represents a reduced number of matches compared to patient details only). *MRN* Medical record number

**Fig. 2:**
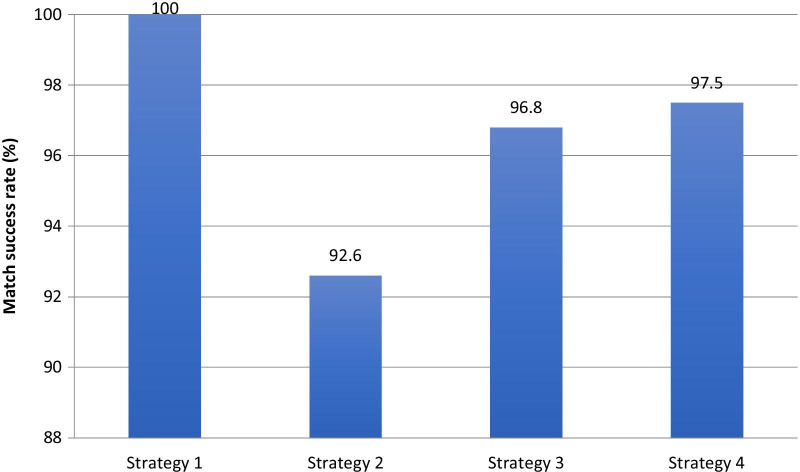
Bar chart showing match rate of different strategies. Strategy 1: ‘gold standard’ using all data available including identified data, plus admission/procedure date. Strategy 2: deidentified data only, plus admission/procedure date. Strategy 3: SLK-581 plus admission/procedure date. Strategy 4: as for strategy 2, plus SLK-581

There were 1273/1283 matching patients using direct identifiers. 10 patients had no possible match to an ICU patient. When the match was restricted to patients in whom the procedure date matched admission date this number reduced to 1242/1273. 1242 patients therefore represented the ‘gold standard’ for these datasets. There were no incorrectly matched patients (false positive matches). Linkage summary statistics are shown in Figs. 1 and 2.

### Strategies 2

**Fig. 3:**
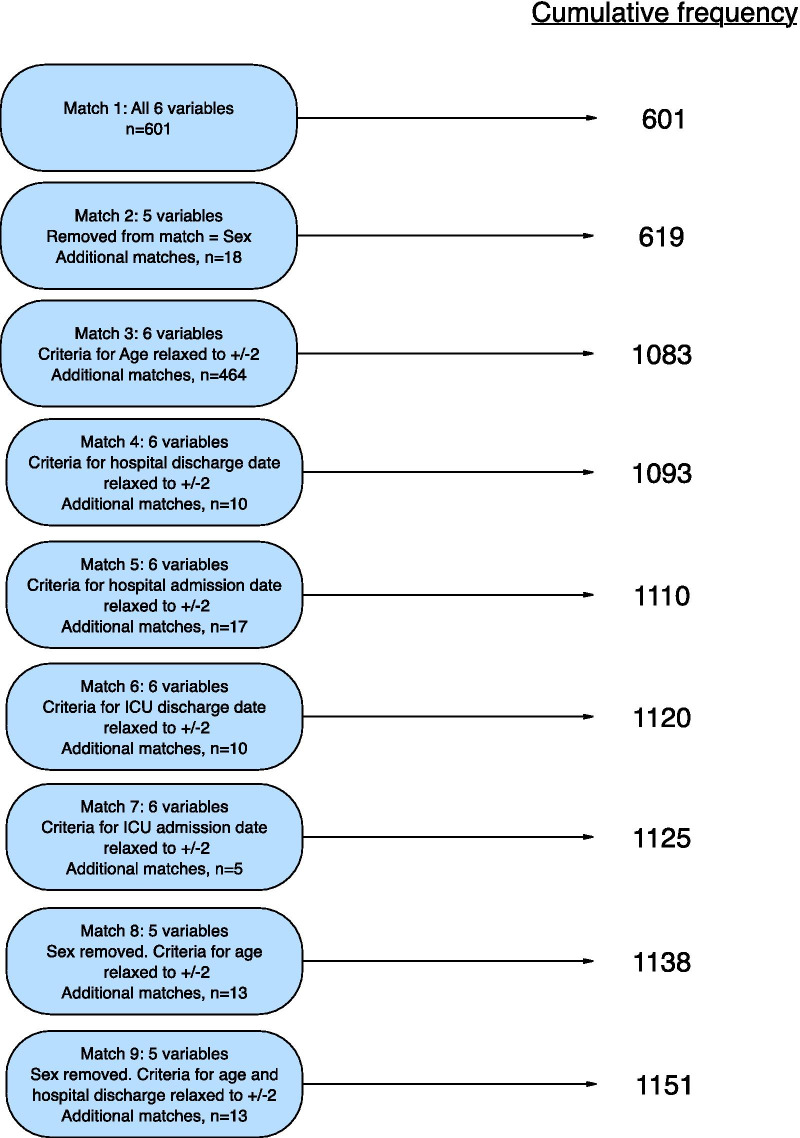
Matching process and results for deidentified variables. The six variables for match 1 are ICU admission date, ICU discharge date, hospital admission date, hospital discharge date, age and sex

Using deidentified data for matching there were 1151/1242 (92.6%) matches. Cumulative percentage linkage using deidentified data is shown in Fig. 3. There were no incorrectly matched patients (false positive matches).

### Strategy 3

Linkage using SLK-581 and procedure date alone yielded 1202/1242 (96.8%) patients. There were no incorrectly matched patients (false positive matches).

### Strategy 4

Matching using deidentified data was improved to 1211/1242 (97.5%) when SLK-581 was added. This 60 patient improvement in match was due to patients matched using SLK-581 and ICU admission date only (data mismatches were in other date fields).

The match rate for SLK-581 was better than for deidentified data alone (1202/1242 vs. 1151/1242, *p* < 0.001). When using SLK-581 the addition of other deidentified fields did not significantly improve the match rate (1211/1242 vs. 1202/1242, *p* = 0.17). No strategy was as successful as using patient identifying information (1242 vs. 1202, 1151 and 1211). Unmatched patients in the ‘gold standard’ (strategy 1) group were younger, required the procedure more urgently, had a higher risk of death and had more ‘non-standard’ cardiac procedures compared to matched patients (Table 2). Characteristics in unmatched patients were similar between the other three strategies, with small differences in risk of death and urgency of procedure. Unmatched patients (representing ‘false negatives’) in strategies 2–4 occurred as a result of missing data or data errors in multiple fields.

**Table 2:** Comparison of characteristics between matched and unmatched patients using strategy 1, and between unmatched patients using all 4 strategies

Strategy 1	Matched patients (n = 1242)	Unmatched patients (n = 41)	p-value
Age (mean, sd)	63.5 (12.7)	53.6 (15.1)	< 0.001^a^
Male sex (%, n)	77.3 (960)	80.5 (33)	0.63^e^
Risk of death % (median, IQR)	1.08 (0.67–2.10)	7.50 (1.33–14.5)	< 0.001^c^
Urgent/emergent procedure (%, n)	22.0 (273)	58.5 (24)	< 0.001^e^
*Procedure type (%, n)*			
CABG	56.6 (703)	26.8 (11)	< 0.001^e^
Valve repair/replacement	15.1 (187)	17.1 (7)	
Combined CABG/Valve	7.49 (93)	4.9 (2)	
Other	20.1 (259)	51.2 (21)	

Unmatched patients:	Strategy 1 (n = 41)	Strategy 2 (n = 132)	Strategy 3 (n = 72)	Strategy 4 (n = 81)	p-value
Age (mean, sd)	53.6 (15.1)	59.7 (14.7)	57.2 (15.7)	57.7 (16.2)	0.15^b^
Male sex (%, n)	80.5 (33)	80.3 (106)	81.9 (59)	80.3 (65)	0.99^e^
Risk of death % (median, IQR)	7.50 (1.33–14.5)	2.36 (1.2–7.6)	4.26 (1.29–13.2)	1.62 (0.82–8.78)	0.02^d^
Urgent/emergent procedure (%, n)	58.5 (24)	43.2 (57)	55.6 (40)	35.8 (29)	0.02^e^
*Procedure type (%, n)*					
CABG	26.8 (11)	29.6 (39)	29.2 (21)	39.5 (32)	0.76^e^
Valve repair/replacement	17.1 (7)	19.7 (26)	16.7 (12)	13.6 (11)	
Combined CABG/Valve	4.9 (2)	5.3 (7)	5.6 (4)	8.6 (7)	
Other	51.2 (21)	45.5 (60)	48.6 (35)	38.3 (31)	

Unmatched patient p-values refer to a comparison between all 4 groups. Comparison uses total available patients in database as denominator (n = 1283)

Tests used as follows:

^a^Student’s t-test

^b^Analysis of variance (ANOVA)

^c^Wilcoxon rank-sum

^d^Kruskall-Wallis

^e^Chi-square

## Discussion

All methods of matching yielded a match rate > 90%. SLK-581 improved the capacity to link data compared to using non-identifiable variables. SLK-581 alone as a merging variable was not significantly improved by the addition of other non-identifying variables. There were differences in patient characteristics between matched and unmatched patients.

This study suggests that the linkage of databases where a complete set of identifying data is not available may be improved by using SLK-581, compared to other non-identifying shared data fields. This situation could occur in 3 instances. Firstly, where both databases contain SLK-581 (for example the ANZICS-APD and the Australasian Rehabilitation Outcomes Consortium (AROC)). Secondly where only one database contains SLK-581 but the other contains full identifying data (e.g. ANZICS-APD and ANZSCTS-CSD). Finally, where both contain full identifying data but for reasons of privacy the full identifying dataset cannot be released to either party.

Other studies have noted variable results as SLK-581 is prone to data errors in the same way as non-identifiers [12]. It is possible matching could be improved if each character in the SLK was treated as a separate entity and matching criteria relaxed to enable small typographical errors to be accounted for. Weber et al., using a linkage key with date of birth and two letters from the surname and first name found similar results, with a 97% sensitivity, improved compared with social security or full names (which were prone to errors). They noted variation on names provided a significant source of error, which was improved by using only the first letters [13]. Kum et al. also note the reduced sensitivity using full names and exact matches [4]. Problems have been noted in studies of larger databases, whereby ‘missed links’, or false negative matches, may occur [9]. For example, where patients have more than one SLK-581 due to alternate spellings or hyphenation. These may be improved by the addition of further information, for example area of residence or postcode [14]. Our linkage had a higher rate of success compared to other studies [8, 12]. This may be due to a much smaller cohort, the use of a single centre’s data, and the high quality of the collected data [15, 16]. In addition, some have raised concerns regarding limited privacy [8, 9]. Given that the key contains a full date of birth and other letters of the surname without encryption it is conceivable that reidentification could occur. Indeed, there are more modern and sophisticated means of preventing unintended patient identification, such as one-way encryption of identifying details. These could be used in concert with linkage keys where no other identification is available. This could provide a higher level of security and reduce the possibility of reidentification where data is particularly sensitive. However, this would have the added effect of preventing a ‘relaxed’ criteria match to account for typographical errors, as encrypted data may be radically different with only small differences in original data. Additionally, some of these processes could be automated to allow easy access to accurate linked data [17].

It is possible that differences in data quality between this and other centres may result in different linkage results. However, data collection is standardised between centres and it appears likely that results would be similar. 10 patients were missing from the ICU database. These could represent data error, or rare cases of no ICU admission, for example death or direct ward admission. There were significant differences between matched and unmatched patients. In particular, the risk of death in unmatched patients was significantly higher, patients were younger and underwent more urgent, non-standard procedures. This probably occurred for two reasons. Firstly, although rare, it is possible that patients may not survive their procedure, and therefore may not have an ICU admission. Secondly, and more commonly, the most unwell patients will be admitted to ICU prior to their procedure occurring and therefore will not have a post procedure admission date to match the procedure date. Given that much of the data from the ANZICS-APD is recorded for the first 24 h from admission, linkage of the initial data (for example, a few days prior to their cardiac surgical procedure) would yield irrelevant matches. This is because the majority of the incremental information from the ANZICS-APD contains acute physiological data. This information changes rapidly and is unlikely to be relevant if not temporally matched to the cardiac surgical procedure. While differences in match rates between different strategies were small overall, scaled up to larger populations they could be significant, particularly if specific patient groups are over-represented in unmatched patients. It was notable that there were no incorrectly matched patients (false positives). This may be due to the ‘clean’ nature of the test data used in this study, and therefore may not apply to other less ‘clean’ datasets. Future work could involve evaluating these methods on synthetic data to which imperfect data has been added.

## Conclusion

We present successful linkage between two registry databases using SLK-581. Linkage was improved compared to using non-identified data, but was not as good as using patient name and unique record number.

## Data Availability

The data that support the findings of this study are available from the ANZSCTS Database and the Alfred Hospital but restrictions apply to the availability of these data, which were used under license for the current study, and so are not publicly available.

## Abbreviations

ANZICS: Australian and New Zealand Intensive Care Society
ANZSCTS: Australian and New Zealand Society of Cardiothoracic Surgeons
APD: Adult patient database
AIHW: Australian Institute of Health and Welfare
CSD: Cardiac surgery database
ICU: Intensive care unit
SLK: Statistical linkage key
MRN: Medical record number
AROC: Australasian Rehabilitation Outcomes consortium
CABG: Coronary artery bypass grafting
